# The CD47/TSP-1 axis: a promising avenue for ovarian cancer treatment and biomarker research

**DOI:** 10.1186/s12943-024-02073-0

**Published:** 2024-08-14

**Authors:** Aurélie Moniot, Christophe Schneider, Laure Chardin, Elisa Yaniz-Galende, Catherine Genestie, Marion Etiennot, Aubéri Henry, Coralie Drelon, Audrey Le Formal, Benoit Langlois, Laurence Venat, Christophe Louvet, Laure Favier, Alain Lortholary, Dominique Berton-Rigaud, Nadine Dohollou, Christophe Desauw, Michel Fabbro, Emmanuelle Malaurie, Coraline Dubot, Jean Emmanuel Kurtz, Nathalie Bonichon Lamichhane, Éric Pujade-Lauraine, Albin Jeanne, Alexandra Leary, Stéphane Dedieu

**Affiliations:** 1 Apmonia Therapeutics, Reims, France; 2 UMR 7369 MEDyC, CNRS, Université de Reims Champagne-Ardenne, Reims, France; 3 Gustave-Roussy Cancer Campus Université Paris-Saclay GINECO/GINEGEPS, Inserm U981, Villejuif, France; 4 Centre Hospitalier Universitaire Dupuytren, Limoges, France; 5 Institut Mutualiste Montsouris-Jourdan, Paris, France; 6 Centre Georges-François Leclerc, Dijon, France; 7 Hôpital Privé du Confluent - GINECO, Nantes, France; 8 ICO Centre René Gauducheau - GINECO, Saint-Herblain, France; 9 Polyclinique Bordeaux Nord, Bordeaux, France; 10 Centre Hospitalier Régional Universitaire de Lille, Hôpital Huriez, Lille, France; 11 ICM Val d’Aurelle - GINECO, Montpellier, France; 12 Centre Hospitalier Intercommunal de Créteil, Créteil, France; 13 Institut Curie - Hôpital René Huguenin - GINECO, Saint-Cloud, France; 14 Hôpitaux Universitaires de Strasbourg, Strasbourg, France; 15 Clinique Tivoli-Ducos, Bordeaux, France; 16 ARCAGY-GINECO, Paris, France

**Keywords:** Ovarian cancer, TSP-1, CD47, PARP, Neoadjuvant chemotherapy

## Abstract

**Background:**

Ovarian cancer (OC) remains one of the most challenging and deadly malignancies facing women today. While PARP inhibitors (PARPis) have transformed the treatment landscape for women with advanced OC, many patients will relapse and the PARPi-resistant setting is an area of unmet medical need. Traditional immunotherapies targeting PD-1/PD-L1 have failed to show any benefit in OC. The CD47/TSP-1 axis may be relevant in OC. We aimed to describe changes in CD47 expression with platinum therapy and their relationship with immune features and prognosis.

**Methods:**

Tumor and blood samples collected from OC patients in the CHIVA trial were assessed for CD47 and TSP-1 before and after neoadjuvant chemotherapy (NACT) and multiplex analysis was used to investigate immune markers. Considering the therapeutic relevance of targeting the CD47/TSP-1 axis, we used the CD47-derived TAX2 peptide to selectively antagonize it in a preclinical model of aggressive ovarian carcinoma.

**Results:**

Significant reductions in CD47 expression were observed post NACT. Tumor patients having the highest CD47 expression profile at baseline showed the greatest CD4^+^ and CD8^+^ T-cell influx post NACT and displayed a better prognosis. In addition, TSP-1 plasma levels decreased significantly under NACT, and high TSP-1 was associated with a worse prognosis. We demonstrated that TAX2 exhibited a selective and favorable biodistribution profile in mice, localizing at the tumor sites. Using a relevant peritoneal carcinomatosis model displaying PARPi resistance, we demonstrated that post-olaparib (post-PARPi) administration of TAX2 significantly reduced tumor burden and prolonged survival. Remarkably, TAX2 used sequentially was also able to increase animal survival even under treatment conditions allowing olaparib efficacy.

**Conclusions:**

Our study thus (1) proposes a CD47-based stratification of patients who may be most likely to benefit from postoperative immunotherapy, and (2) suggests that TAX2 is a potential alternative therapy for patients relapsing on PARP inhibitors.

**Supplementary Information:**

The online version contains supplementary material available at 10.1186/s12943-024-02073-0.

## Background

**Fig. 1:**
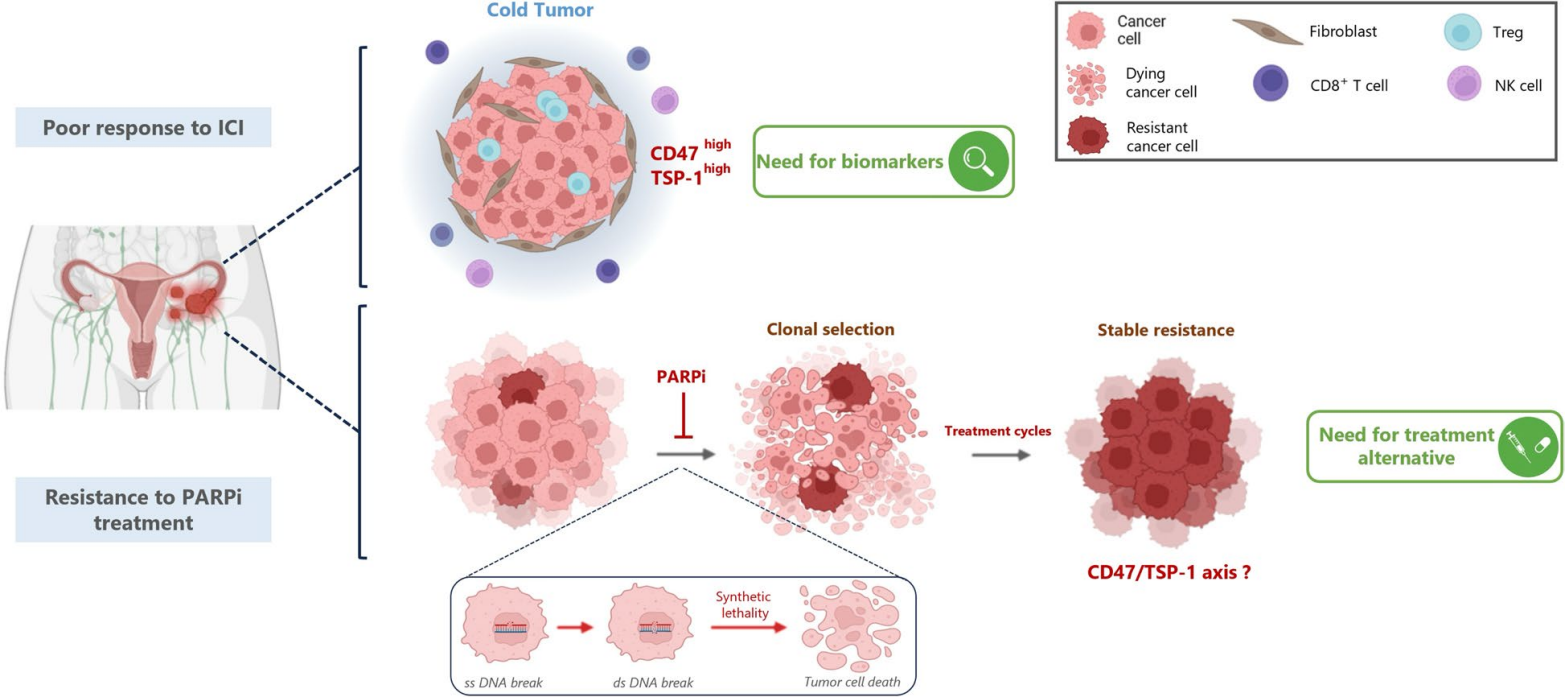
Schematic illustration of two major clinical challenges in ovarian carcinoma. The lack of therapeutic response to immune checkpoint inhibitors (ICI, top panel). There is no treatment for patients who relapse after treatment with PARPi (bottom panel)

Ovarian cancer (OC) ranks as the eighth most prevalent cancer in women worldwide, with nearly 314,000 new cases and 207,252 deaths documented in 2020 [1]. Early diagnosis significantly improves the prognosis. However, only 20% of OC cases are identified at stage I, and the 5-year relative survival rate plunges to 17% for patients diagnosed with stage IV [2]. For advanced-stage disease, the standard of care is cytoreductive debulking surgery and platinum-based chemotherapy, with potential incorporation of angiogenesis inhibitors [3–5]. The emergence of poly-ADP ribose polymerase (PARP) inhibitors has transformed the landscape of OC management in recent years [6, 7]. Despite these substantial advances, OC management still faces significant challenges, as depicted in Fig. 1. First, the immunosuppressive tumor microenvironment makes OC refractory to immunotherapies targeting PD-1/PD-L1 (Fig. 1, top panel), despite numerous trials testing combinatorial strategies aimed at re-activating the immune response [8–10]. A deeper understanding of the intricate immune tumor microenvironment (iTME) and the impact of chemotherapy on immune features holds promise in unveiling novel immune targets and potentially reshaping therapeutic strategies [11, 12]. Second, resistance to PARP inhibitors (PARPis) is one of the most important and immediate challenges facing oncologists today (Fig. 1, bottom panel). At least half of OC tumors are homologous recombination deficient (HRD), and PARPi maintenance has been shown to significantly improve survival for women with HRD OC [6, 13–15]. Unfortunately, many of these patients fail to respond, or they relapse during PARPi treatment, and demonstrate poor outcomes with subsequent chemotherapy, probably due to overlapping resistance mechanisms [16, 17]. Addressing both these challenges will require a better understanding of the unique tumor immune landscape of OC and the intricate mechanisms triggering PARPi resistance, along with the emergence of reliable biomarkers that are easy to use in clinical trials [5].

Amidst these challenges, CD47 is emerging as a key player, being widely expressed in OC and potentially contributing to immune evasion by interacting with circulating ligands, notably thrombospondin-1 (TSP-1). While most studies have focused on the CD47/signal regulatory protein (SIRP)α interface (the “don’t eat me” signal) [18], the CD47/TSP-1 axis is gaining attention in the field of oncology because it influences tumor cell survival [19] and cancer stem cell differentiation [20], supports chemotherapy and radiotherapy resistance [21], and inhibits the anti-cancer immune response [22–26]. In line with our vision, a recent review challenges the prevailing CD47/SIRPα dogma by suggesting that TSP-1 is the likely source of immune signaling via CD47 in trans, while SIRPα may contribute to the regulation of CD47 in cis and trans [27]. TAX2, a cyclic peptide derived from CD47, shows promise in selectively antagonizing TSP-1 binding to CD47 [28–32]. Xenograft and allograft preclinical mouse models have demonstrated TAX2 efficacy in targeting OC growth, stimulating anti-tumor immunity, and acting synergistically with anti–PD-1 immune checkpoint inhibitors while maintaining a favorable safety profile [23].

However, little is known about how the expression of the CD47 receptor or its ligand TSP-1 change under chemotherapy, or about their association with other features of iTME. In the randomized CHIVA trial, we therefore investigated changes in CD47 expression in OC patient paired samples, from diagnosis to post-neoadjuvant chemotherapy (NACT), and assessed the relationship of CD47 expression to other iTME features, including circulating levels of TSP-1. Additionally, using murine models we evaluated whether inhibiting the CD47/TSP-1 axis could overcome PARPi resistance in OC.

## Methods

### CHIVA trial

As previously described, the double-blind randomized phase II GINECO-sponsored CHIVA trial (NCT01583322) evaluated the benefit of neoadjuvant carboplatin plus paclitaxel (NACT) with or without nintedanib in 188 women with stage III/IV epithelial OC. The trial result was negative, showing no benefit in terms of progression-free survival [33]. However, after informed consent from participating patients, paired formalin-fixed, paraffin-embedded (FFPE) tumor samples were obtained at diagnosis and after 3 cycles of NACT in order to study changes in iTME during NACT. As previously published, there was no difference in anti-tumor activity or impact on iTME between treatment arms. Thus, tumors were analyzed regardless of treatment arm. The methods adhered to pertinent guidelines and regulations, and the study received approval from ANSM on June 6, 2012. Additionally, it received a favorable opinion from the local medical ethics committee Ile de France 1 on March 29, 2012. All patients provided written consent for participation in translational research sub-studies.

### Immunostaining

FFPE samples were selected based on the greatest viable tumor cellularity; necrotic samples were excluded. In a few patients with complete pathological response, immune parameters post NACT were not evaluated. The presence of tumor cells was confirmed by H&E staining followed by review by an expert pathologist. The most representative and relevant regions of each tumor were chosen after pathology review to construct the tissue microarray (TMA). Three 1.2-mm cores from each tumor sample were used to construct the TMAs. Chromogen-based IHC analysis was performed on the 3 μm–thick TMA sections for the detection of CD47 using the Automated Discovery Ultra staining system (Ventana Medical Systems, Roche). Briefly, all TMA sections were deparaffinized at 72 °C using the EZ Prep, heat pre-treated at 95 °C for 68 min in cell conditioning medium II for antigen retrieval, incubated at room temperature (RT) with DISC inhibitor medium for endogenous peroxidase inactivation, and incubated at RT for 60 min with the rabbit anti–human CD47 antibody (Abcam, clone SP279, 1:50). The antibody used for CD47 staining was a commercially available one, previously validated for IHC on FFPE samples [34], for which the exact epitope is proprietary information. As such we cannot determine whether the antibody detected all CD47 isoforms or only certain post-translational modified variants. CD47 expression was scored by H-score using QuPath software: staining intensity (0, + 1, + 2, + 3) × % cells positive (0 to 100) = 0 to 300. Multiplex immunofluorescence staining (CD4, CD8, FOXP3, CD68, CD163) and image analysis were conducted as previously described [12].

### Plasma TSP-1 quantification

Whole blood was collected in K2 EDTA tubes in a subset of patients (*n* = 59). Plasma was separated from cellular components of the blood, aliquoted, and stored at − 80 °C until analysis. Blood samples were centrifuged at 1000 × *g* and 4 °C for 10 min, then the plasma supernatant was transferred to a new vial and subjected to a second round of centrifugation at 10,000 × *g* and 4 °C for 10 min to ensure the removal of all cellular components. Circulating TSP-1 was quantified by ELISA on paired plasma samples pre and post NACT using a Human Thrombospondin-1 Quantikine ELISA kit (Catalog no. DTSP10, R&D Systems, MN, USA), according to the manufacturer’s protocol.

### TAX2 peptides

TAX2 was manufactured by PolyPeptide Laboratories (Malmö, Sweden) using Fmoc-SPPS as already described [23]. TAX2 was stored at − 20 °C and extemporaneous solubilization was performed in PBS. The TAX2-Cy5 peptide was produced by click chemistry (Genepep, France), stored at − 20 °C and put at room temperature before solubilization in 6.25% DMSO in injectable saline solution. Quality control testing was conducted using ultra high-performance liquid chromatography and mass spectrometry. Peptide stability and solubility were checked before use.

### Microscale thermophoresis

Microscale thermophoresis (MST) analysis was performed using a NanoTemper Monolith NT.115 Pico apparatus (NanoTemper Technologies, Cambridge, MA). Recombinant human TSP-1 (R&D Systems, 3074-TH) was labeled with a Monolith His-Tag kit RED-tris-NTA 2nd generation (NanoTemper Technologies, Cambridge, MA). Labeled TSP-1 (10 nmol) was incubated for 20 min at room temperature in the dark with a serial dilution testing series of TAX2 peptide (from 2.5 × 10^−4^ M to 7.63 × 10^−9^ M) in PBS, 0.1% Tween 20. Samples were loaded into standard Monolith NT.115 capillaries, and MST analysis was performed (medium MST power, excitation 20% with Pico-RED excitation type). Recorded data were analyzed using MO.Affinity Analysis software v2.3 (NanoTemper Technologies).

### Animal care and ethics committee approval

C57BL/6 J female mice were purchased from Janvier Labs (Le Genest-Saint-Isle, France) or Charles River Laboratories (Saint-Germain Nuelles, France). Animals were fed a standard laboratory diet with water and food given ad libitum and kept under constant environmental conditions. All studies were performed in compliance with the French Animal Welfare Act and the requirements of the French Board for Animal Experiments. Experiments were conducted under the approval of the French Ministère de l’Enseignement Supérieur et de la Recherche (ethics committees n°C2EA-56) in compliance with Directive 2010/63/UE under the direction of investigators certified for animal experiments following APAFIS#28592–2020111014534200 v4, APAFIS#21989–2018102515497191 v15, and APAFIS#29756–2020111014474011 v10.

### Biodistribution of TAX2-Cy5 in healthy mice

Experiments were carried out on 8-week-old C57B/6 J mice weighing 19–22 g. Two weeks before TAX2-Cy5 injection, mice were fed with food without chlorophyll. The back and abdomen were depilated just before experiment initiation. Mice (2–6 per group) received an intravenous (IV) bolus dose (1 to 20 mg/kg BW) of TAX2-Cy5 peptide. At a defined time point after TAX2-Cy5 injection, mice were anesthetized and placed in a portable animal imaging cassette. Whole-body fluorescence was then quantified, with instrument sensitivity set to “Normal” using laser channel 635 of a fluorescence molecular tomograph (FMT) 4000 (Perkin Elmer). Blood samples were taken under isoflurane anesthesia using intracardiac sampling. Blood samples were collected on EDTA tubes and then spun at RT 10 min at 2000 g (minimal brake). Plasma samples were twofold diluted in NaCl 0.9%, then the fluorescence count in each sample was determined with instrument sensitivity set to “Normal”. After mouse euthanasia, the kidneys, lungs, spleen, heart, liver, stomach, brain, and bladder were collected and weighed, then the fluorescence count in each sample was determined with instrument sensitivity set to “Normal”. The region of interest was determined on samples and backgrounds using an ellipsoid on each subject. Background was subtracted from each sample using the software operation and fluorescence count was then determined. TAX2-Cy5 concentration in tissues was calculated from the fluorescence count (pmol) in each sample adjusted for the volume of tissues. TAX2-Cy5 concentration in tissues was compared to TSP-1 gene (*THBS1*) expression based on the Human Protein Atlas database, combining data from three transcriptomics datasets (HPA, GTEx, and FANTOM5) and using the internal normalization pipeline.

### Biodistribution of TAX2-Cy5 in tumor-bearing mice

A suspension of ID8 *Trp53*^*−/−*^* Brca*^*−/−*^ mouse ovarian carcinoma cells (kindly provided by Professor Iain McNeish, Imperial College London, UK) was inoculated in C57B/6 J mice either subcutaneously (SC, *n* = 3) into the left flank or intraperitoneally (IP, *n* = 2) into the left side (5 × 10^6^ cells in 300 µL HBSS per animal). After 5 (IP model) or 10 (SC model) weeks of tumor growth, mice received an IV bolus dose of TAX2-Cy5 at 10 mg/kg BW in injectable saline solution. After in vivo imaging and mouse euthanasia, subcutaneous tumor or peritoneal tumor mass and metastatic nodules, were collected and imaged with instrument sensitivity set to “Normal” using the same protocol as described above for healthy mice. TAX2-Cy5 concentration was calculated as described above.

### Evaluation of TAX2 efficacy alone or in sequential combination with olaparib

Eight-week-old C57BL/6 J mice were IP injected with 2 × 10^6^ ID8 *Trp53*^*−/−*^* Brca2*^*−/−*^ cells in 300 µL HBSS. For mouse treatments, TAX2 peptide was IV injected at 30 to 100 mg/kg, three times a week; olaparib (catalog no. M1664, AbMole, TX, USA) was given per os at a 50 mg/kg BW dose, daily. Two different sequential treatments were performed. In the first one, mice were treated one day after tumor inoculation with olaparib for 2 weeks followed by 4 weeks of TAX2. In the second one, mice were randomized 7 days after tumor inoculation and first treated with olaparib daily for 5 weeks or an identical volume of olaparib vehicle composed of 10% (2-hydroxypropyl)-β-cyclodextrin and 10% DMSO in D-PBS buffer. Mice were then sequentially treated with TAX2 for a maximum of 3 weeks. During the treatment periods, no sign of toxicity was observed in the drug-treated mice whatever the doses being considered. Mice were weighed twice or thrice a week and abdominal distension was measured using a digital caliper. Tumor burden was monitored using body weight change as a measure of ascitic fluid buildup post inoculation as previously described in the same model [35]. Gain of 20% body weight was considered as the end point for survival analysis. In this orthotopic model, it is impossible to determine whether the absence of tumor uptake is treatment related or not. Therefore, mice showing no signs of tumor development (carcinosis, nodules, or ascites) at the time of euthanasia were excluded to avoid misinterpretation of the results.

### Statistics

Two-tailed Pearson and Spearman tests were used to assess the correlation between two linear or monotonic variables, respectively, and Mann–Whitney test was used where appropriate. Mouse survival curves were generated using Kaplan–Meier analysis with Gehan–Breslow–Wilcoxon tests as previously reported for ID8 syngeneic model [36, 37]. All graphs and statistics were generated with Prism (GraphPad Software, La Jolla, CA) using the statistical tests and sample sizes indicated in the figure legends.

## Results

### Neoadjuvant chemotherapy leads to decreased CD47 expression in ovarian carcinoma

**Fig. 2:**
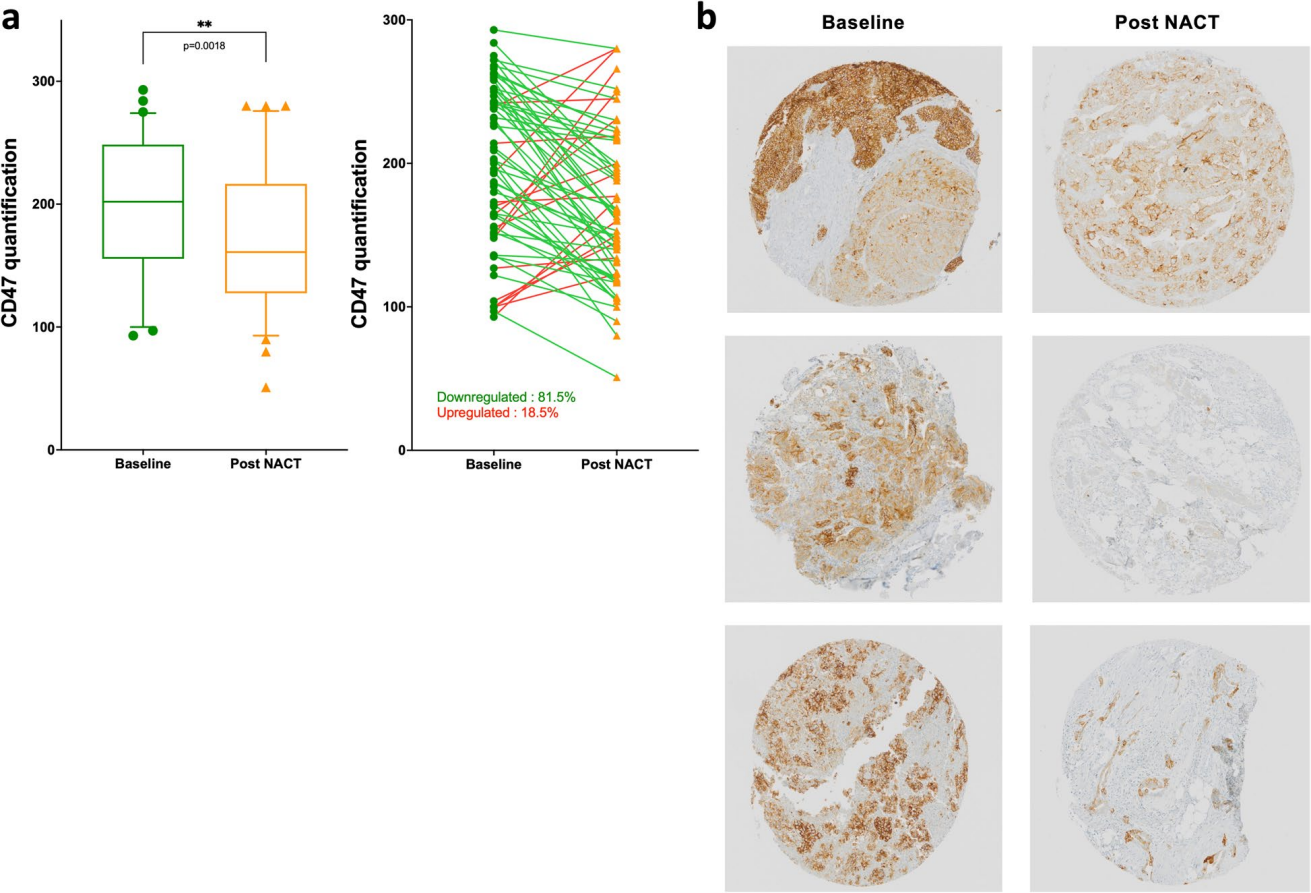
Immunohistochemical assessment of CD47 expression in ovarian carcinomas at diagnosis and after neoadjuvant chemotherapy. CD47 expression was assessed by IHC in paired samples at diagnosis (baseline, green point) and after neoadjuvant chemotherapy (NACT) (orange triangle) (*n* = 65). The quantification was conducted using the H-score system, combining staining intensity (0, + 1, + 2, + 3) with the percentage of positive cells (0 to 100) for a score of 0 to 300. **a** Lines connect samples from the same patient and indicate an increase (red) or decrease (green) in CD47 expression (left panel). Histograms showing quantification of CD47 expression (right panel). Statistical significance is indicated as **p < 0.01 (two-tailed Mann–Whitney test). **b** Representative images displaying representative CD47 staining patterns in three tumors from OC patients before and after NACT

CD47 expression was assessed by IHC in paired samples at the time of diagnosis and following 3 cycles of NACT and scored by H-score (Suppl Fig. 1a). As depicted in Fig. 2a and b, the initial expression of CD47 was high (median value of 202), with no cases being completely negative for CD47 expression. Using the median as a cutoff, high CD47 expression at baseline was associated with improved overall survival (OS) (Supp Fig. 1b). There was a significant downregulation in CD47 levels in paired samples after NACT (median H-score: 161 vs 202, *p* = 0.0018, Fig. 2a). When considering changes in individual patients, most patients (81.5%) demonstrated decreasing tumor CD47 expression post NACT (Fig. 2b).

### CD47 levels correlate with CD8 and CD4 infiltration after NACT only in the population with highest CD47 expression

**Fig. 3:**
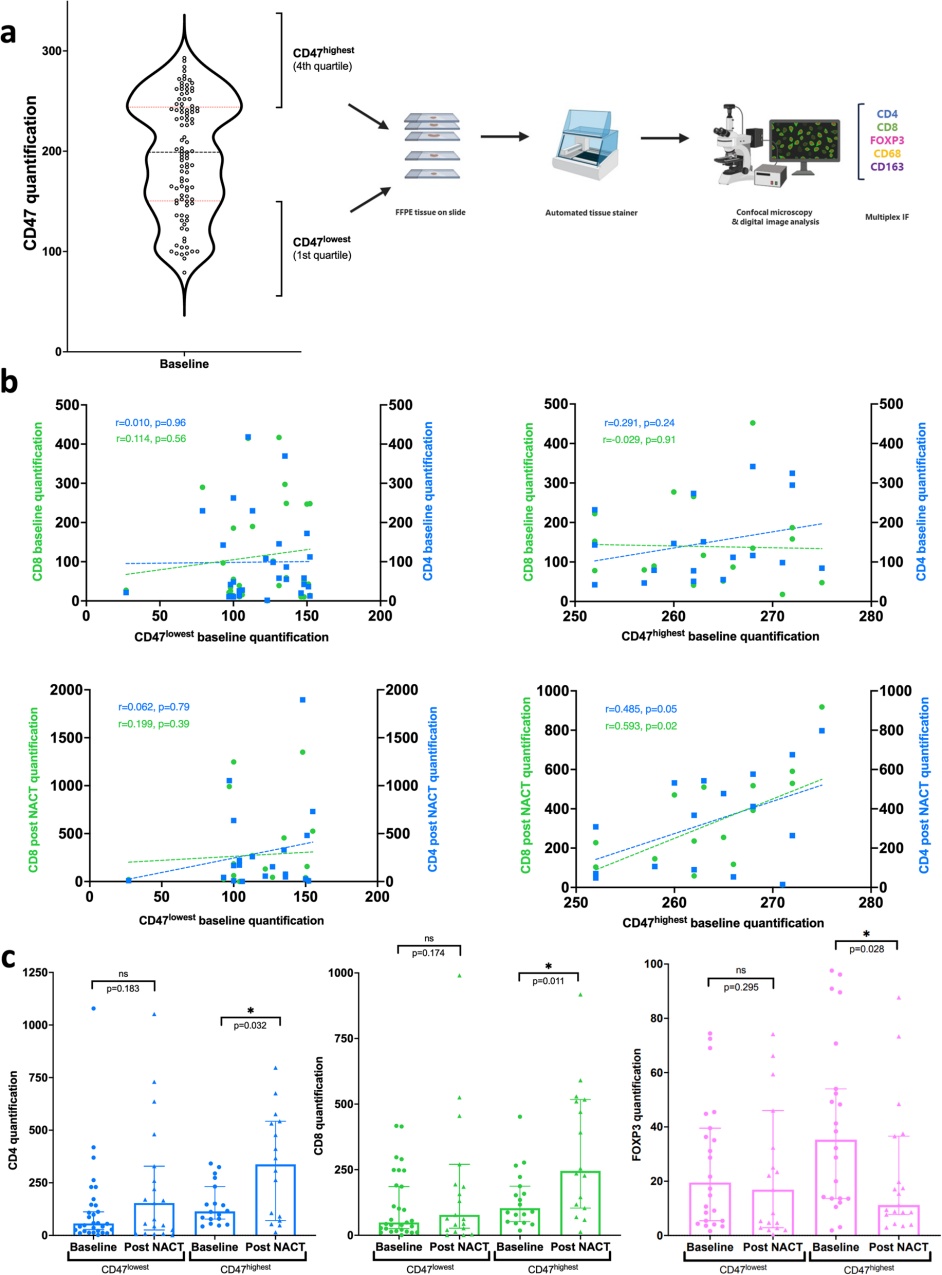
(previous page). Correlation of CD47 with immune features after NACT. Tumor samples were collected from 101 patients from the CHIVA trial. **a** At baseline, we defined a CD47^highest^ (fourth quartile, score above 240) and a CD47^lowest^ population (first quartile, score under 150). Tumor samples were collected from 101 patients from the CHIVA trial. CD4^+^, CD8^+^, CD68^+^, CD163^+^, and FoxP3^+^ cells were assessed by immunofluorescence and scored as the number of positive cells. A mean score was calculated from three TMA cores from each sample. **b** CD8 (green point) and CD4 (blue square) staining were quantified at baseline (upper panels) or post NACT (lower panels) in the CD47^lowest^ (left panels) and CD47^highest^ (right panels) populations. The Pearson correlation coefficient (r) is indicated on each panel. The *p*-value was determined using the Wald test. **c**, Histogram representing CD4^+^ (blue), CD8^+^ (green), and FOXP3^+^ (pink) signal intensities at baseline (circle) and after NACT (triangle) in the CD47^lowest^ and CD47.^highest^ populations. Statistical significance is indicated as ns (not significant) or *p < 0.05 (one-tailed Mann–Whitney test)

To discern the impact of varying CD47 expression, we categorized tumors into CD47^highest^ and CD47^lowest^ quartiles according to their CD47 expression levels (Fig. 3a). Tumors obtained from these 2 subpopulations underwent thorough multiplex analysis to investigate immune markers. While CD47^highest^ did not correlate with CD4 and CD8 expression at baseline (Fig. 3b, top right panel), a correlation with CD8 (*r* = 0.59, *p* = 0.02) and a trend with CD4 (*r* = 0.49, *p* = 0.05) emerged after NACT (Fig. 3b, bottom right panel). Conversely, these linear correlations were absent within the CD47^lowest^ population (Fig. 3b, left panels). Furthermore, in the CD47^highest^ group, a significant increase in CD4^+^ cell (threefold, *p* = 0.032) and CD8^+^ cell (2.4-fold, *p* = 0.011) tumor infiltration was observed after NACT (Fig. 3c). This sharply contrasts with the CD47^lowest^ group, where no significant change was observed (Fig. 3c, left and middle histograms). Additionally, NACT induced a remarkable 2.9-fold decrease in FOXP3^+^ cells, only within the CD47^highest^ group (*p* = 0.028) (Fig. 3c, right histogram). Consequently, both the CD4^+^/ FOXP3^+^ (*p* = 0.001) and CD8^+^/FOXP3^+^ (*p* = 0.0005) ratios exhibited a twofold increase post NACT, exclusively in OC patients displaying the highest CD47 expression (Suppl. Fig. 2a and Suppl. Fig. 3). Interestingly, these associations appear T-cell specific since no correlation exists when considering CD68 and CD163 before or after NACT, for either the CD47^highest^ or the CD47^lowest^ subset (Suppl. Fig. 2b and Suppl. Fig. 4). This stratification based on CD47 expression significantly delineated overall survival outcomes, with a favorable prognosis among patients exhibiting the highest CD47 expression at diagnosis (Suppl. Fig. 2c).

### Plasma TSP-1 Levels in OC patients: prognostic implications and response to NACT

**Fig. 4:**
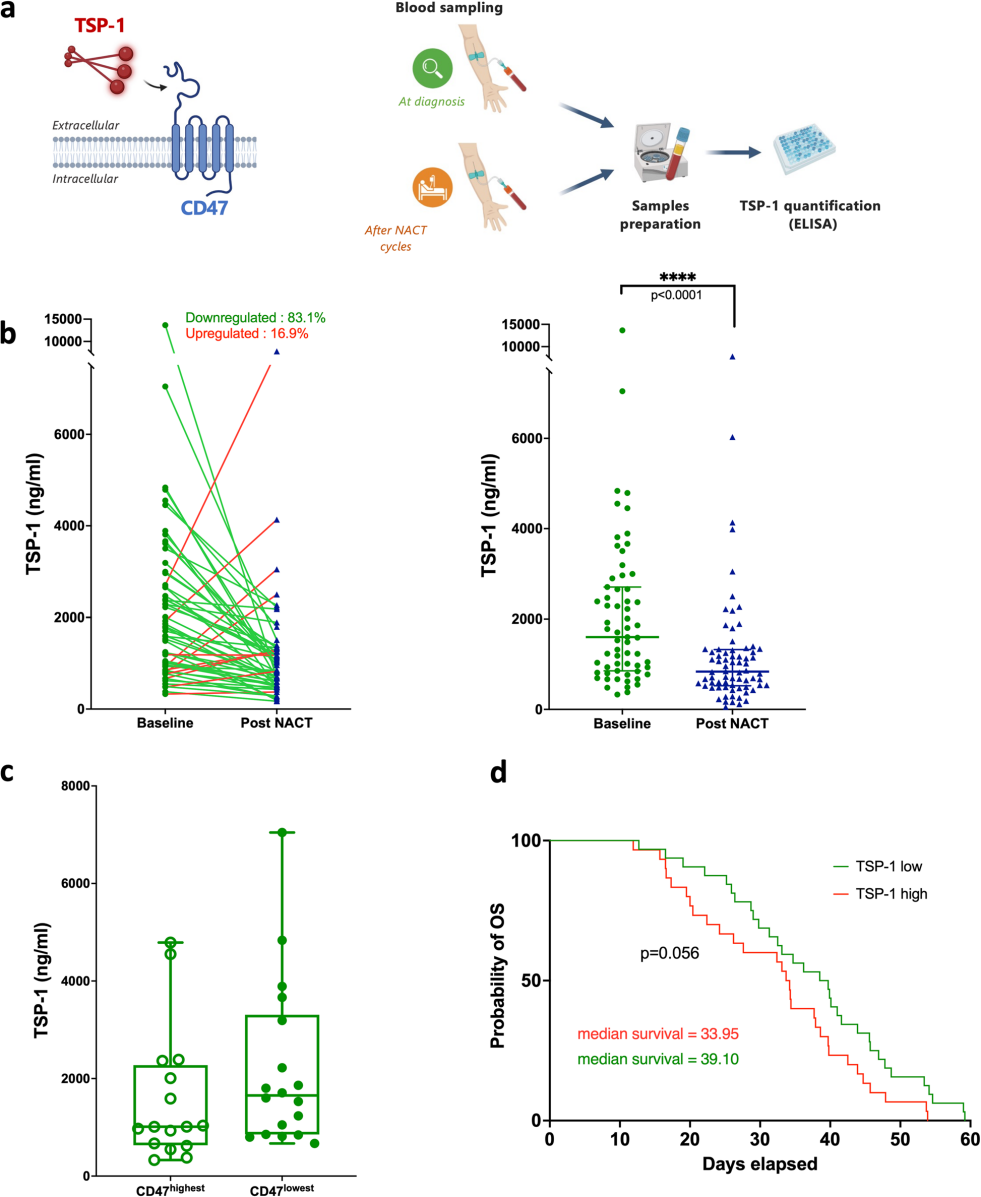
Circulating TSP-1 is decreased after NACT. **a** Schematic illustration depicting the functional interaction between the CD47 receptor and the TSP-1 glycoprotein (left panel). The strategy employed to measure circulating TSP-1 levels in OC patients is depicted in the right panel. **b** Plasma levels of TSP-1 were determined using paired samples (*n* = 59) from the CHIVA cohort at baseline (green point) and post NACT (blue triangles). Connective lines (red for increased, green for decreased) link samples from the same patient to illustrate changes after treatment. Statistical significance is indicated by ****p < 0.0001 (two-tailed Mann–Whitney test). **c** Correlation plot displaying plasma TSP-1 levels versus tissue CD47 expression. **d** Kaplan–Meier survival curve representing patient overall survival (OS) based on circulating TSP-1 levels. The median was used as a cutoff to discriminate between low and high TSP-1 expression

In ovarian carcinogenesis, the matrix glycoprotein TSP-1 plays a significant role as a primary ligand interacting with CD47 (Fig. 4a, left panel). Despite prior observations of tissue TSP-1 overexpression in ovarian tumors [23, 38], its discriminatory potential remains limited based on current knowledge. This is further complicated by conflicting results concerning the correlation between tissue expression levels of TSP-1 and patient survival [39]. Therefore, our focus shifted to investigating the secreted and circulating form of TSP-1, quantified using plasma samples obtained pre and post chemotherapy (Fig. 4a, right panel). As shown in Fig. 4b, the plasma concentration of TSP-1 was found to be high (> 1500 ng/mL) in OC patients. Intriguingly, 83.1% of patients showed a decrease in TSP-1 post NACT. Analysis of the entire cohort revealed an approximate twofold decrease in circulating TSP-1 levels following NACT (*p* < 0.0001). Notably, albeit based on a limited sample size, a trend emerged wherein the highest levels of circulating TSP-1 were found in patients with CD47^low^ tumors at diagnosis (Fig. 4c), those tumors also showing the least response to NACT in terms of lymphocyte immune response (see Fig. 3). Consistently, lower overall patient survival correlated with elevated plasma TSP-1 levels (Fig. 4d).

### TAX2 is a suitable tool for targeting the TSP-1/CD47 pathway in preclinical models of OC

**Fig. 5:**
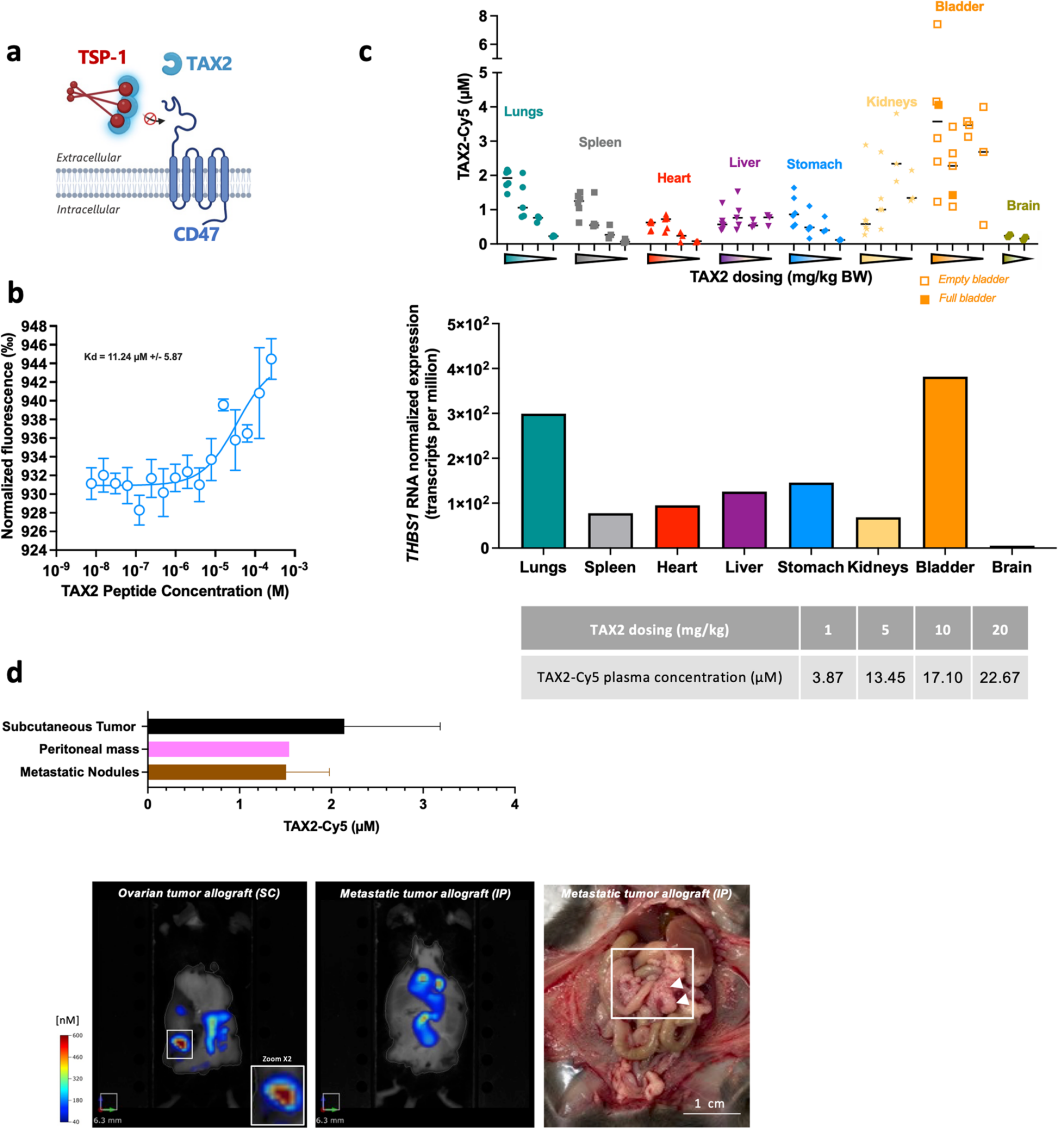
TAX2: binding, pharmacokinetics, and biodistribution studies. **a** Schematic diagram illustrating the mechanism of action of TAX2, which binds the carboxy-terminal domain of TSP-1, selectively inhibiting TSP-1/CD47 interaction. **b** Graph displaying the dose–response curve for the binding between TAX2 and labeled rhTSP-1 (10^−8^ M) as assessed by microscale thermophoresis. **c** Evaluation of TAX2 biodistribution in healthy C57BL/6 J mice using FMT after IV administration of a dose range of TAX2-Cy5 (1, 5, 10, or 20 mg/kg, 2–6 mice/group). Fluorescence quantification was performed on collected tissues at T0. The middle part of panel c presents the TSP-1 gene (THBS1) expression in various human tissues obtained from the Human Protein Atlas database, combining datasets from HPA, GTEx, and FANTOM5 as of April 2024. The table (lower part of panel) presents fluorescent quantification on plasma collected at T0. **d** Assessment of TAX2-Cy5 biodistribution (administered IV at a 10 mg/kg BW dose) in mice bearing ID8 Trp53^−/−^ Brca2.^−/−^ ovarian carcinoma cells injected either subcutaneously (SC) or intraperitoneally (IP). Fluorescence quantification using FMT was conducted on live mice after 24 h (bottom panel). Subsequently, subcutaneous tumors (black bar, SC model), peritoneal tumor masses, and metastatic nodules (pink and brown bars, respectively, IP model) were excised post euthanasia and then imaged for TAX2-Cy5 quantification. Necropsy (bottom panel, right image) reveals metastases in the omentum (highlighted by white arrows)

Given the significant unmet medical need of OC progression during PARPi treatment (refer to Fig. 1, bottom section), we further explored the potential significance of targeting the CD47/TSP-1 axis in this context. TAX2, a 12-amino acid cyclic peptide derived from CD47 [28], is the only existing compound that selectively targets TSP-1 and disrupts TSP-1 binding to CD47 (Fig. 5a). MST analysis indicated that TAX2 peptide binds directly to human TSP-1 with K_D_ of 11 µM (Fig. 5b). Biodistribution studies of TAX2 peptide were conducted in both healthy mice (Fig. 5c) and mice bearing ovarian tumors (Fig. 5d), using fluorescent labelling tracked through FMT. Remarkably, a dose-dependent study in healthy mice displayed a peptide distribution pattern in organs driven by its target expression (Fig. 5c). Of note, TAX2 organ distribution did not lead to accumulation in the kidneys or liver, even with increasing doses. TAX2-Cy5 fluorescent signal was also quantified from plasma samples and a trend toward proportionality between dose and plasma concentration was observed (Fig. 5c). In addition, we demonstrated the localization of TAX2 at tumor sites in mice bearing ovarian tumors (Fig. 5d).

### Evaluation of TAX2 efficacy in OC, including post–PARP inhibitor treatment

**Fig. 6:**
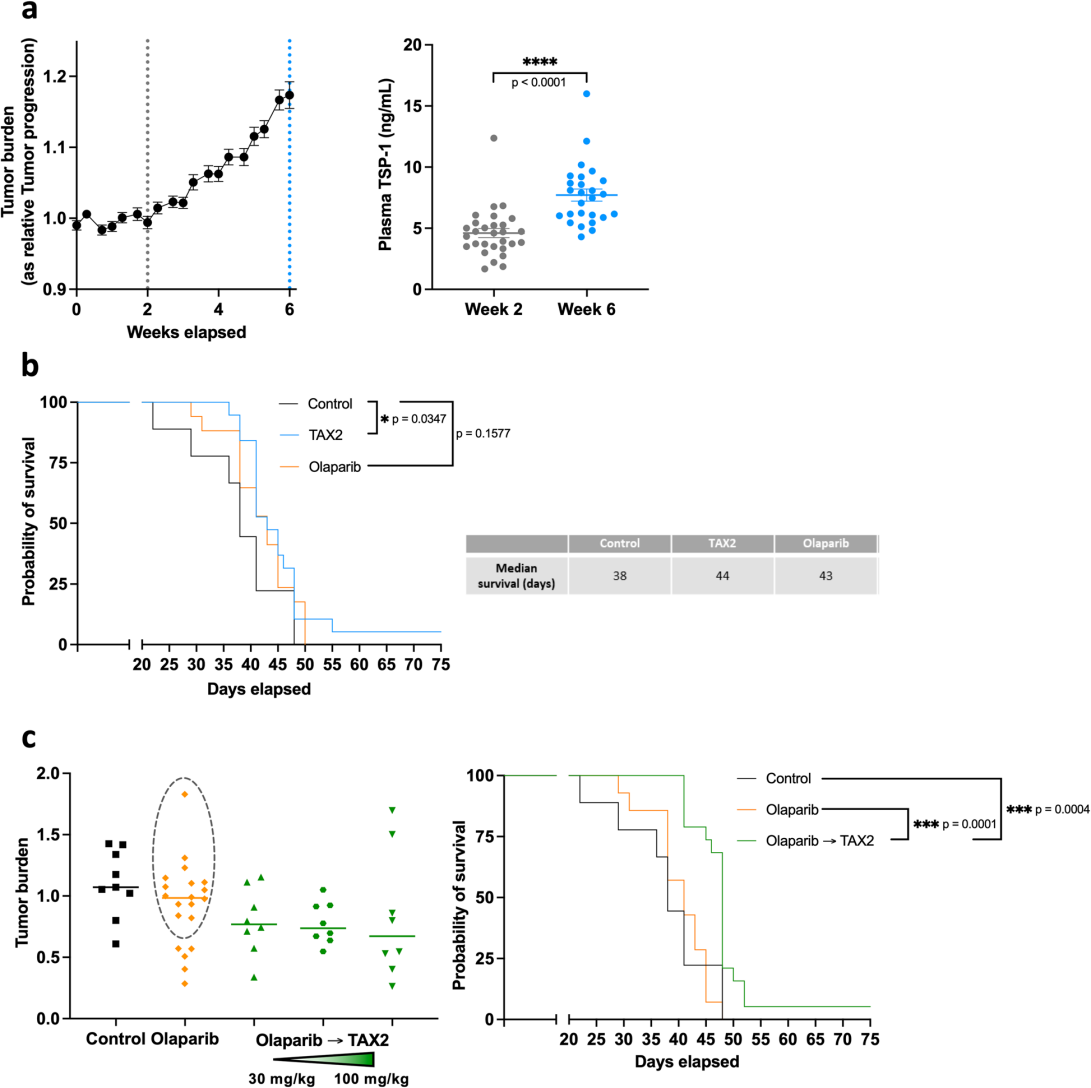
TAX2 efficacy in a metastatic mouse model of ovarian cancer. **a** A metastatic ovarian carcinoma model was established in C57BL/6 J female mice by IP injection of 2 × 10^6^ ID8 Trp53^−/−^ Brca2^−/−^ cells. Tumor burden was monitored by assessing the change in relative body weight as a result of intense ascites production over 6 weeks post inoculation with tumor cells. Plasma TSP-1 concentration was quantified using an ELISA assay. **b** C57BL/6 J female mice (*n* = 9–19 per group) were IP inoculated with ID8 Trp53^−/−^ Brca2^−/−^ cells and treated the following day with either olaparib (50 mg/kg, per os, daily for 2 weeks) or TAX2 (30 mg/kg, IV, 3 times weekly for 6 weeks). Kaplan–Meier overall survival analysis illustrates the outcome among control mice (black), olaparib (orange), and TAX2 treated mice (blue). Survival curves were compared using the Gehan–Breslow–Wilcoxon test as previously described [37]. The table presents median survival for each treatment group. **c** C57BL/6 J female mice were IP injected with ID8 Trp53^−/−^ Brca2.^−/−^ and treated with vehicle (*n* = 9), olaparib alone (50 mg/kg, per os, daily for 2 weeks, *n* = 20) or sequentially with olaparib (50 mg/kg, per os, daily for 2 weeks) followed by TAX2 (30 to 100 mg/kg, IV, 3 times weekly for 4 weeks) (*n* = 8 per TAX2 dosing). The left panel indicates the tumor response, with the dotted black circle representing mice unresponsive to olaparib treatment. The right panel presents Kaplan–Meier overall survival analysis comparing control mice (black, *n* = 9), olaparib-resistant mice (orange, *n* = 14), and mice treated sequentially with olaparib then TAX2 (30 mg/kg, green, *n* = 19), using the Gehan–Breslow–Wilcoxon test. *p < 0.05, ***p < 0.001

To assess the efficacy of TAX2 and/or PARPi treatment, we used ID8 cells harboring *Trp53*^*−/−*^ and *Brca2*^*−/−*^ mutations; these cells exhibit accelerated tumor growth rates as compared with wild-type ID8. This metastatic allograft model serves as a highly relevant syngeneic representation of intraperitoneal high-grade serous *TP53*- and *BRCA2*-mutated ovarian carcinoma, suitable for evaluating PARPi (olaparib) efficacy [40]. Change in mouse body weight was used to track intraperitoneal tumor burden as already reported [35], given its strong correlation with ascites volume (*r* = 0.67, *p* < 0.0001) (Suppl. Fig. 5, left panel). Conversely, sagittal diameter measurements were found to be a less reliable indicator of tumor load (Suppl. Fig. 5, middle panel). The exponential phase of tumor burden was characterized from week 2 following intraperitoneal tumor cell inoculation (Fig. 6a, left panel). Interestingly, the plasma TSP-1 level exhibited a 1.7-fold increase during the phase of tumor expansion (Fig. 6a, right panel). Olaparib treatment failed to improve animal survival, confirming PARPi resistance in this model (Fig. 6b), whereas administration of TAX2 alone modestly but significantly extended the survival rate compared with that of the controls (*p* = 0.0347). Specifically, the median survival increased to 44 days post TAX2 treatment, compared with 38 days in the control group. Notably, 1 out of 9 mice treated with TAX2 showed a weak peritoneal carcinomatosis (Suppl. Fig. 6) and survived for the duration of the experimental protocol (Fig. 6b), a phenomenon not observed in the groups receiving the vehicle or olaparib treatment.

To mimic the clinical challenge of finding a therapeutic alternative when PARPi resistance occurs, we established a sequential treatment protocol. TAX2 administration therefore followed treatment with olaparib and the onset of resistance. Data from Fig. 6c (left panel) indicated that 75% of mice exhibited no response to olaparib, resulting in a median tumor burden identical to that of the control arm. Remarkably, mice subjected to sequential treatment with olaparib followed by TAX2 showed reduced tumor progression even at the lowest TAX2 dosing (Fig. 6c, left panel and Suppl. Fig. 7), correlating with an improved animal survival rate (Fig. 6c, right panel; *p* = 0.0001 vs olaparib treatment). Consequently, the median survival significantly increased to 48 days as compared with survival in the control (38 days) and olaparib-resistant mice (41 days).

**Fig. 7:**
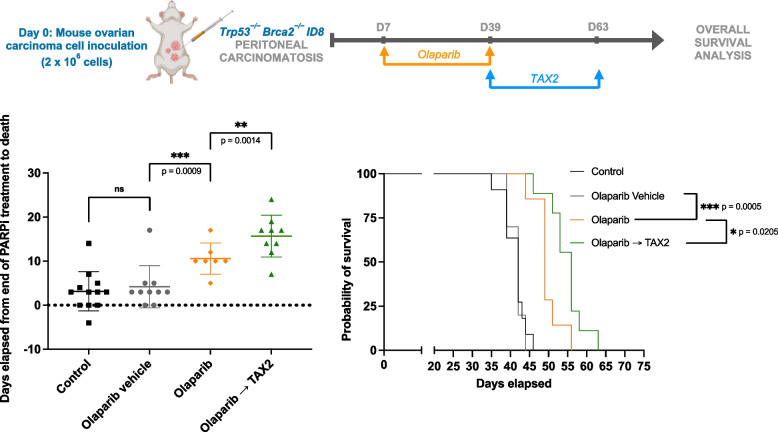
Efficacy of a sequential combination of olaparib with TAX2 in a peritoneal carcinomatosis model. ID8 Trp53^−/−^ Brca2.^−/−^ carcinoma cells were IP injected into mice as described in Fig. 6 to induce peritoneal carcinomatosis. At day 7 post inoculation, animals were randomly assigned (*n* = 7–11 per group) and treated with olaparib for 5 weeks (per os, daily at 50 mg/kg) or an equivalent volume of olaparib vehicle. Subsequently, mice were sequentially treated with TAX2 until the end of the protocol (IV, three times a week at 30 mg/kg). Kaplan–Meier overall survival analysis comparing control mice (black), olaparib-vehicle mice (gray), olaparib-treated mice (orange), and mice treated sequentially with olaparib then TAX2 (green) is presented. Statistical analysis was performed using the Gehan–Breslow–Wilcoxon test, denoted as *p < 0.05 and ***p < 0.001

Since only 2 weeks of PARPi alone did not result in a significant anti-tumor effect, we next implemented an experimental design involving a more prolonged olaparib exposure lasting 5 weeks (Fig. 7). More prolonged olaparib exposure increased mouse survival in this setting, and sequentially administered TAX2 further improved survival. Post olaparib discontinuation, mouse survival increased with administration of TAX2 (Fig. 7, bottom-left panel, *p* = 0.0014) and this resulted in improved median survival (Fig. 7, bottom-right panel, *p* = 0.0205). Thus, TAX2 demonstrated its efficacy in improving survival rates when administered sequentially, irrespective of the duration of prior PARPi exposure.

## Discussion

The results of our study highlight several essential features of the CD47/TSP-1 axis within the OC immune microenvironment in response to NACT and document the potential of targeting this axis in the challenging post-PARPi setting.

We first observed that CD47 is ubiquitously and highly expressed in OC patients at diagnosis and is associated with improved overall survival. Our results seem to be at odds with previous investigations reporting an association between CD47 expression and poor clinical-pathological parameters in OC [41–43] and endometrial cancers [44]. This correlation is not corroborated by our findings regarding OS. Our results shed new light in a context where the status of CD47 as a prognostic biomarker remains debated, notably due to the lack of clinical data. Intriguingly, platinum-resistant patients exhibit enhanced CD47 expression [45], suggesting its potential as a predictive marker for platinum resistance. Our results therefore provide nuance to previous research highlighting the negative impact of CD47 in various cancers, including OC. CD47 is indeed reported to foster epithelial ovarian tumor cell growth, to promote OC progression and metastasis, and to facilitate immune evasion by impeding macrophage phagocytosis [46, 47].

Contrary to other studies reporting that chemotherapy induces upregulation of CD47 in several cancer indications [48], we observed a marked decrease in CD47 expression after NACT in most OC patients. Limited attention has been devoted to exploring the role of CD47 in immune infiltration in OC. Our data suggest that OC patients exhibiting the highest CD47 expression profile at baseline have the greatest lymphocyte influx post NACT, potentially rendering them the most responsive to postoperative immunotherapy. We have previously reported that NACT can have immunomodulatory properties favoring immune surveillance [12, 49]. For the first time, we have shown that NACT also significantly decreases both CD47 as well as its circulating ligand TSP-1. Importantly, those tumors exhibiting the highest CD47 levels at baseline demonstrate improved survival and the most favorable immune response to NACT, with significant influx of CD8^+^ and CD4^+^ T-cells post NACT. Tumors with the highest CD47 expression also had decreased numbers of FOXP3^+^ T-cells, resulting in a favorable cytotoxic to regulatory T-cell ratio post NACT in favor of anti-tumor immunity. In contrast, an inverse relationship was observed with the ligand TSP-1, with those tumors exhibiting high TSP-1 showing poor OS and low influx of cytotoxic CD4^+^ and CD8^+^ T-cells post NACT. We thus clarify an association recently reported between elevated CD47 mRNA expression and immune regulation in OC obtained using KEGG and GSEA analyses [43]. Of note, CD47 expression in our cohort did not predict lymphocytic infiltration at diagnosis, nor macrophagic infiltration before or after NACT. These results shed new light on the expression of CD47 by giving it a valuable informative role in guiding post-NACT therapy.

The emergence of anti-CD47 therapies aimed at impeding CD47 interaction with SIRPα to improve macrophage-mediated cancer cell clearance has attracted much attention in recent years, with monoclonal antibodies in clinical trials [18]. Given the predominance of CD47 positivity in advanced ovarian tumors at diagnosis, with a paucity of negative tumors, CD47 expression does not appear to be a discriminatory inclusion criterion, nor a tool for stratifying patients for anti-CD47 therapy. However, as in many other studies, we are unable to determine whether we are correctly detecting all forms of CD47, including that after pyroglutamate formation at the SIRPα-binding site [50].

In this context, we advocate for plasma TSP-1 as a promising biomarker that supplements the assessment of CD47 status. While tissue overexpression of this extracellular matrix glycoprotein has been linked to OC and associated with advanced stage and poor prognosis [23, 38], it lacks discriminatory power. Additionally, contradictory results have suggested that patients with advanced OC with high TSP-1 staining may experience improved survival [39]. Few studies have explored the biomarker potential of plasma TSP-1, typically focusing on its expression within the primary tumor. Our investigation involving paired plasma samples obtained pre and post chemotherapy unveiled elevated plasma levels of TSP-1 at diagnosis, with a median value around 1500 ng/ml, while recent quantitative proteome profiling data indicate a median level at least two times lower in normal samples [51]. We found that elevated baseline plasma TSP-1 levels correlated with poorer survival outcomes yet decreased under chemotherapy in over 80% of patients, resulting in a two-fold global decrease post treatment. This appears contradictory to findings reporting that higher initial TSP-1 concentrations correlate with improved survival in OC patients [52]. It is worth noting, however, that this previous study quantified TSP-1 in serum, not plasma, and reported median TSP-1 levels significantly lower (118 ng/ml) than those observed in our study. Previous findings indicating an association between high plasma TSP-1 and poor response to paclitaxel in PDX ovarian models emphasize the potential of plasma TSP-1 as a predictive indicator of OC treatment response [53]. Complementarily, our results suggest a tendency towards a correlation between elevated plasma TSP-1 levels and an immune-suppressive profile characterized by low CD47 expression at baseline. Consistent with this, our previous investigation demonstrated that targeting TSP-1 in an orthotopic syngeneic model of metastatic peritoneal carcinomatosis triggers an anti-cancer adaptive immune response synergistic with anti–PD-1 therapy, resulting in enhanced tumor regression and reduced ascites fluid production [23].

Overcoming resistance to anti-PARP therapies remains a major challenge in the management of OC. Interestingly, PARP inhibition was recently reported to upregulate CD47 expression in vitro in OC cells [54]. However, we challenge the relevance of CD47 as a pharmacological target of interest in the context of OC, as we found no correlation between its expression and macrophage infiltration following chemotherapy. While CD47 blockade strategies in recent years have mainly focused on anti-CD47 antibodies, many concerns exist regarding hematotoxicity and off-target effects associated with this strategy due to the widespread expression of CD47 [18, 27]. By way of example, Gilead’s anti-CD47 mAb magrolimab was granted breakthrough drug designation in 2020 and later put on hold by the FDA due to severe side effects in clinical trials [55]. Concomitantly, ALX Oncology in August 2023 announced an end to its ASPEN-02 and ASPEN-05 programs, which were evaluating the efficacy of its CD47 inhibitor evorpacept in myelodysplastic syndrome and acute myeloid leukaemia. More recently, Gilead announced discontinuation of the Phase 3 ENHANCE-3 study, thereby ending development of magrolimab for blood cancer due to increased risk of death [56]. To mitigate these effects, and given the pivotal role of TSP-1 in OC as discussed above, we opted for TAX2 treatment to target the CD47/TSP-1 axis. Toxicology and safety pharmacology studies conducted in Sprague–Dawley rats and beagle dogs revealed that TAX2 is very well tolerated after systemic administration (data not shown). The safety profile of TAX2, if confirmed in clinical trials, would help to overcome the barriers of CD47-targeting approaches. The binding activity of TAX2 to TSP-1 may seem moderate, with a K_D_ in the micromolar range. Of note, this value is likely underestimated by the challenge of replicating in vitro the conditions required to expose the TAX2 binding sequence, embedded in a hydrophobic pocket within the carboxyterminal domain of TSP-1 [57].

Here, we chose to assess TAX2 efficacy in a relevant syngeneic model of high-grade serous OC carrying both *Tp53* and *Brca2* deletions. Remarkably, TAX2 displayed a selective biodistribution pattern in mice, localizing at ovarian tumor sites. Our findings revealed that TAX2 treatment demonstrated notable, albeit moderate, activity in PARPi-naive mice in this particularly aggressive model of metastatic OC, corroborating previous studies performed in different OC xenograft models [23], and other tumor indications [28–32]. Importantly, we observed a significant enhancement in TAX2 efficacy following exposure to a PARPi, regardless of olaparib treatment duration. Importantly, TAX2 treatment initiation does not necessitate waiting for the onset of PARPi resistance or escape mechanisms. This constitutes the first documentation of the association between TSP-1 targeting and the effectiveness of a PARPi. It is a thrilling discovery that introduces novel pharmacological avenues, although the precise mechanisms underlying this synergy remain to be identified. Existing evidence suggests that PARPis enhance T cell-mediated anti-tumor infiltration, and TAX2 similarly operates through distinct molecular pathways [23]. Furthermore, macrophages treated with a PARPi displayed a pro-inflammatory phenotype marked by increased expression of IL-1β [58]. It is anticipated that inhibition of the TSP-1/CD47 signal by TAX2 would similarly promote IL-1β production, as previously documented [26], with IL-1β potentially serving as a shared mediator. Interestingly, while olaparib led to cytokine alterations within the iTME with a notable decrease in IFNγ levels in the ID8 *Trp53*^*−/−*^* Brca2*^*−/−*^ model [40], TAX2 exhibited a contrasting effect, favoring tumor regression [23]. This may help explain, at least in part, how these two compounds complement each other to yield an improved therapeutic effect. There is also a clinical rationale for combining a PARPi with anti-angiogenic drugs in advanced epithelial OC [59]. Interestingly, TAX2 has demonstrated the ability to reprogram highly vascularized tumors into poorly angiogenic ones across various cancer contexts [28, 30–32]. This suggests that TAX2 may synergize with PARPis via both immunogenic and anti-angiogenic mechanisms.

While recent data have demonstrated the synergy between anti-CD47 and PARPi in OC models exhibiting *BRCA* mutation [54], it is worth noting that this study was conducted in xenograft models using immunodeficient mice. This approach may overlook the crucial role of the CD47/TSP-1 axis in the anti-cancer immune response. The authors proposed that inducing the STING pathway may be a mechanism of synergy under PARP inhibition. This may indeed be related to CD47 targeting but seems to date to be dissociated from a TSP-1–dependent signal. Additionally, the combinatorial treatment proposed in this previous study [54] contrasts with our sequential treatment approach, which positions TAX2 in the post-PARPi setting, an area of significant unmet medical need. Using homologous recombination–proficient ovarian tumor cells, another recent study demonstrated that olaparib improved macrophage-associated phagocytosis of cancer cells, with synergy when used in combination with anti-CD47 antibodies [60]. This is consistent with the ability of PARPis to reprogram tumor-associated macrophages toward higher cytotoxicity and phagocytosis [61].

## Conclusions

In conclusion, our study proposes a stratification based on CD47 expression level at diagnosis to identify patients most likely to benefit from postoperative immunotherapy. Our findings also lead us to propose TAX2 as a promising option for PARP inhibitor–relapsed OC patients, with plans underway for a first-in-human trial in this population.

### Supplementary Information

Additional file 1: Suppl. Figure 1. a, Illustration depicting the CD47 staining intensity levels (+ 1, + 2, + 3) used in determining the H-score for CD47 expression assessment. b, Kaplan–Meier survival curves illustrating progression-free survival (PFS) and overall survival (OS) based on CD47 expression level at baseline, categorized as high (red) and low (green). Statistical significance was determined using the log-rank (Mantel–Cox) test for survival differences.

Additional file 2: Suppl. Figure 2. a, Histograms display the CD4+/FOXP3+ ratio (left panel) and CD8+/FOXP3+ ratio (right panel) at baseline (circle) and after NACT (triangle) within the CD47^lowest^ and CD47^highest^ populations. Significance levels are denoted as ns (not significant), **p < 0.01, or ***p < 0.001 based on two-tailed Mann–Whitney tests. b, Quantification of CD68 (orange point) and CD163 (purple square) fluorescence at diagnosis (upper panels) and after NACT (lower panels) in the CD47^lowest^ (left panels) and CD47^highest^ (right panels) populations. Corresponding Pearson correlation coefficients (*r*) and *p*-values derived from Wald tests are indicated for each panel. c, Kaplan–Meier survival curves (PFS and OS) based on baseline CD47 expression levels in the CD47^lowest^ (green) and CD47^highest^ (red) populations (log-rank (Mantel–Cox) test).

Additional file 3: Suppl. Figure 3. T-cell lymphocyte distribution in CD47-positive tumors. Representative images of CD4^+^, CD8^+^, and FOXP3^+^ populations in the CD47^highest^ and CD47^lowest^ populations in pre-NACT (a) and post-NACT (b) tumors.

Additional file 4: Suppl. Figure 4. Macrophage distributions in CD47-positive tumors. Representative images of CD68^+^ and CD163^+^ populations in the CD47^highest^ and CD47^lowest^ populations in pre-NACT (a) and post-NACT (b) tumors.

Additional file 5: Suppl. Figure 5. ID8 Trp53−/− Brca2−/− ovarian carcinoma cells were IP injected into C57BL/6 J female mice as reported in Fig. 6. Correlations between the volume of ascites and the body weight change (left panel) or the sagittal abdominal diameter (middle panel), and between the body weight change and the sagittal abdominal diameter (right panel), were investigated. The linear correlation coefficient r (Spearman) is calculated for each panel.

Additional file 6: Suppl. Figure 6. C57BL/6 J female mice were IP inoculated using ID8 *Trp53*^*−/−*^* Brca2*^*−/−*^ ovarian carcinoma cells, and then treated with vehicle (control, black), olaparib (orange), or TAX2 (blue) as detailed for Fig. 6. Individual tumor burden is represented. Dotted lines represent TAX2 (blue) or olaparib (orange) treatment duration. Mice without carcinosis at sacrifice were eliminated as described in the experimental procedure.

Additional file 7: Suppl. Figure 7. Additional data illustrating the experiment described in Fig. 6c, 6 weeks after inoculation with tumor cells and administration of TAX2 at a 30 mg/kg BW dose, including photographs of mice showing abdominal distension

## Data Availability

The data supporting the findings of this study are not openly available due to reasons of sensitivity and privacy (CHIVA clinical trial), but are available from the corresponding author upon reasonable request.

## Abbreviations

FFPE: Formalin-fixed paraffin-embedded
HRD: Homologous recombination deficient
FMT: Fluorescence molecular tomography
iTME: Immune tumor microenvironment
MST: Microscale thermophoresis
NACT: Neoadjuvant chemotherapy
OC: Ovarian cancer
OS: Overall survival
PARP: Poly-ADP ribose polymerase
SIRP: Signal regulatory protein
TMA: Tissue microarray
TSP: Thrombospondin
