# Supplementary figures and images for: The CD47/TSP-1 axis: a promising avenue for ovarian cancer treatment and biomarker research
Source: Mol Cancer. 2024 Aug 14;23:166. doi: 10.1186/s12943-024-02073-0 (PMC11323699; doi:10.1186/s12943-024-02073-0)

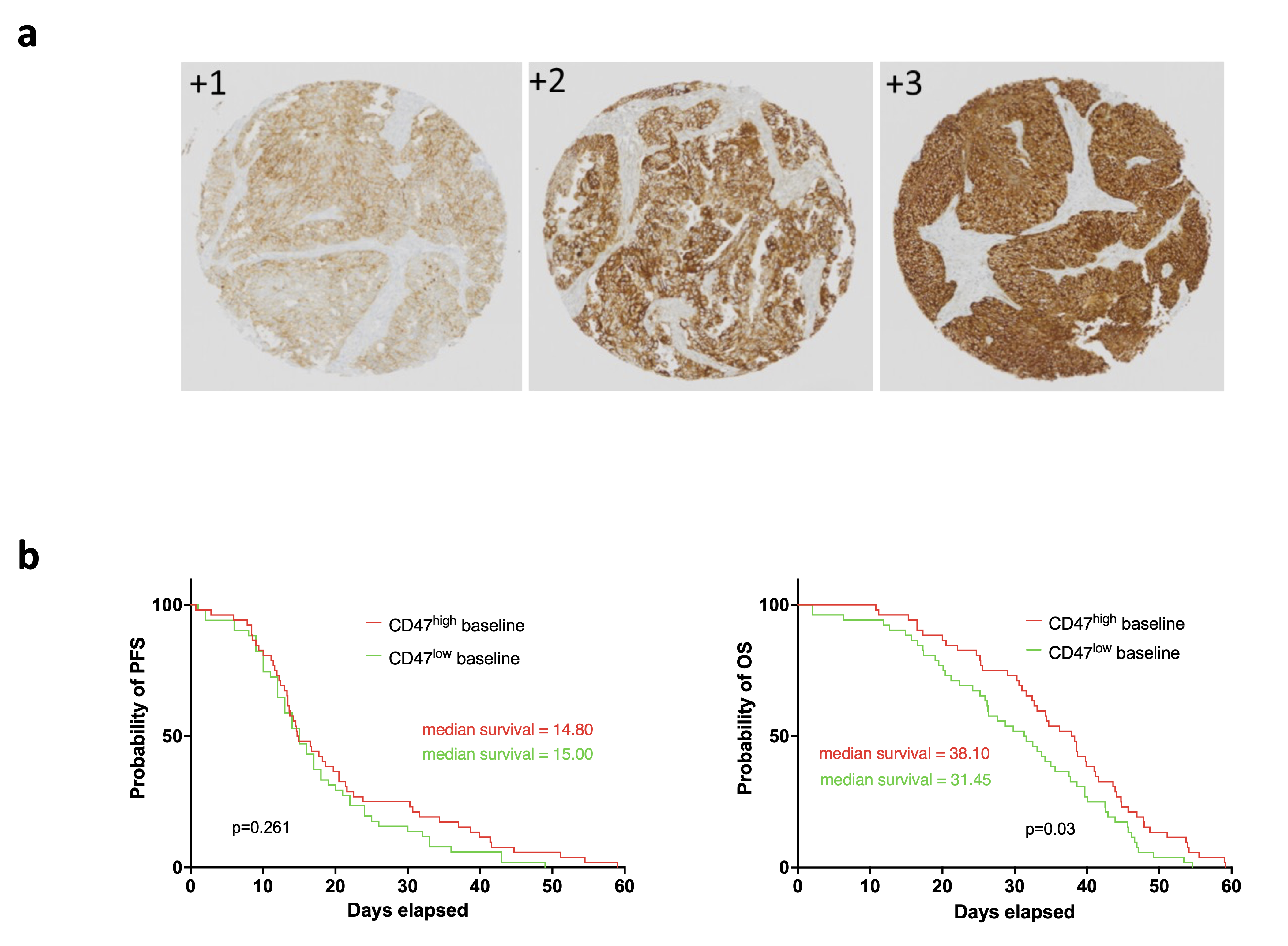

Supplement: Supplementary file 1 — Additional file 1: Suppl. Figure 1. a, Illustration depicting the CD47 staining intensity levels (+ 1, + 2, + 3) used in determining the H-score for CD47 expression assessment. b, Kaplan–Meier survival curves illustrating progression-free survival (PFS) and overall survival (OS) based on CD47 expression level at baseline, categorized as high (red) and low (green). Statistical significance was determined using the log-rank (Mantel–Cox) test for survival differences. [file 12943_2024_2073_MOESM1_ESM.tiff]

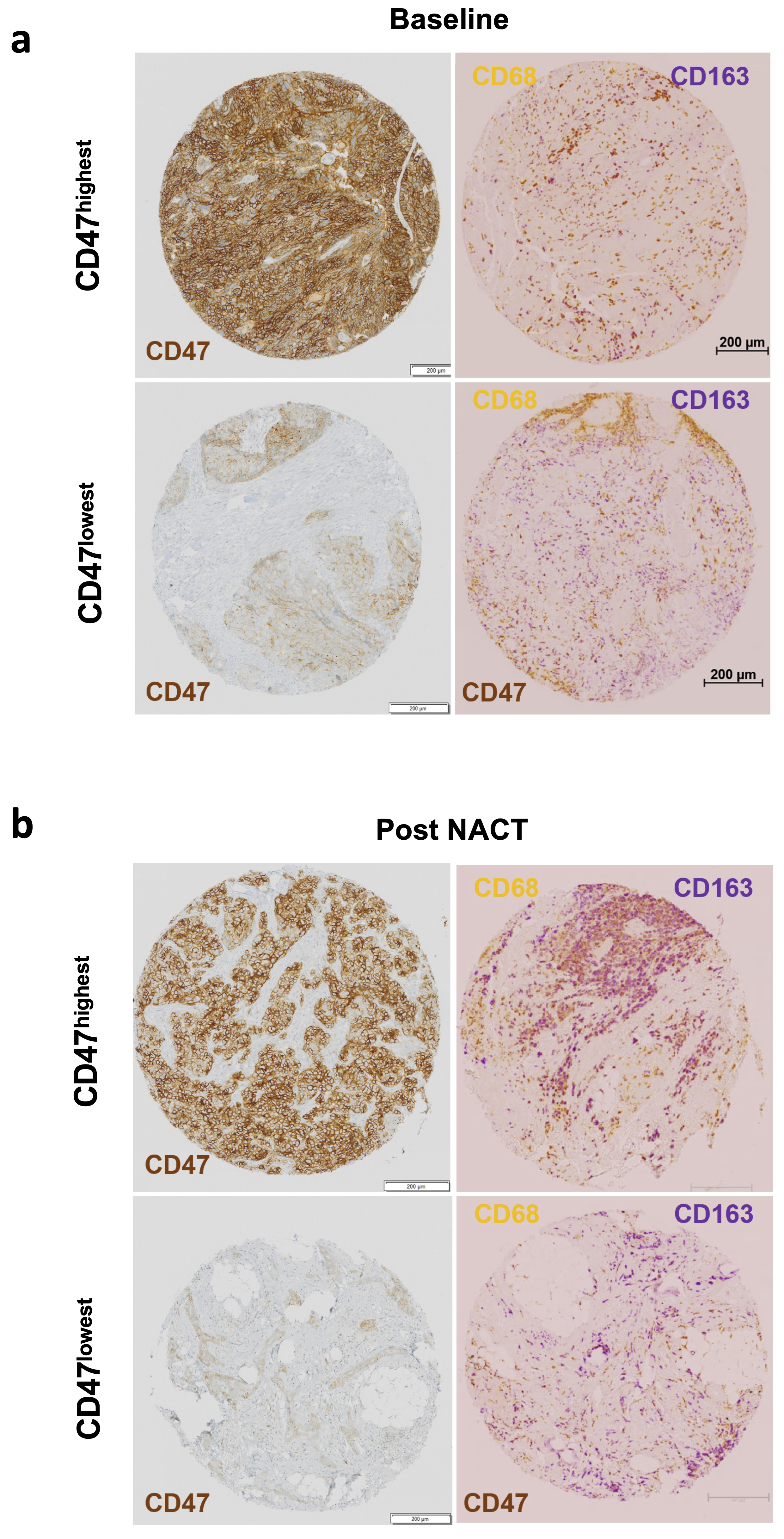

Supplement: Supplementary file 4 — Additional file 4: Suppl. Figure 4. Macrophage distributions in CD47-positive tumors. Representative images of CD68+ and CD163+ populations in the CD47highest and CD47lowest populations in pre-NACT (a) and post-NACT (b) tumors. [file 12943_2024_2073_MOESM4_ESM.tiff]

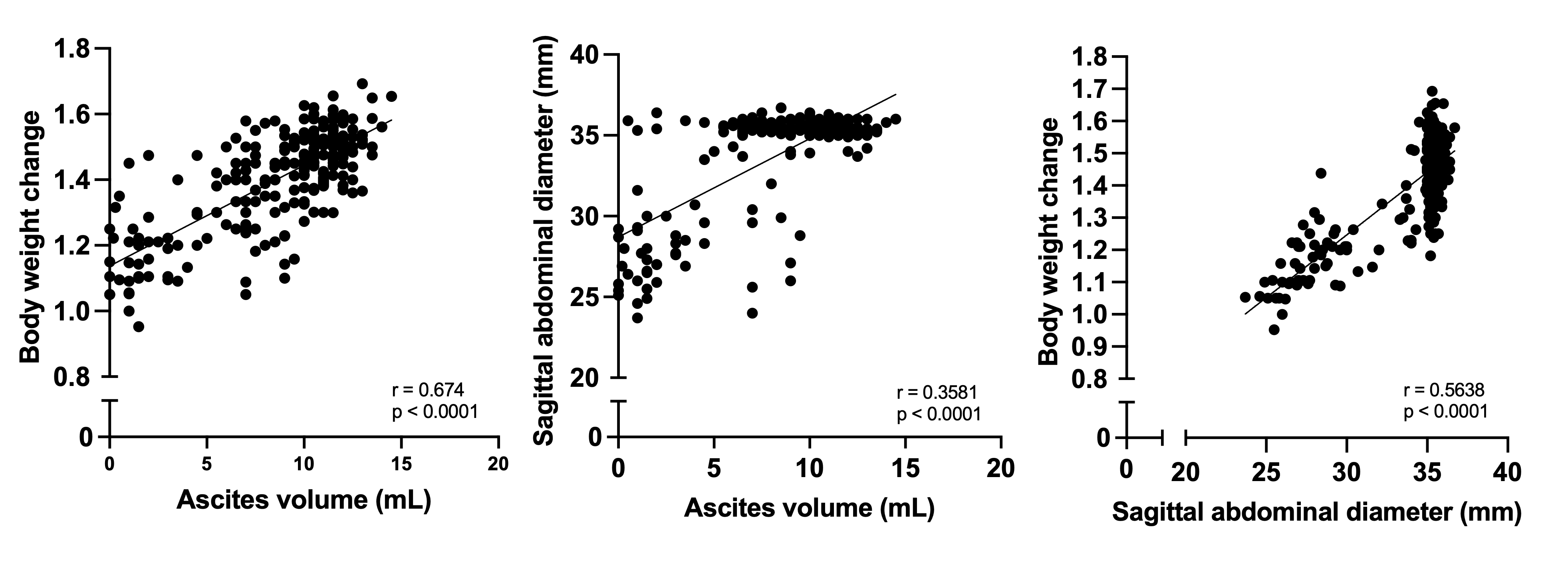

Supplement: Supplementary file 5 — Additional file 5: Suppl. Figure 5. ID8 Trp53−/− Brca2−/− ovarian carcinoma cells were IP injected into C57BL/6 J female mice as reported in Fig. 6. Correlations between the volume of ascites and the body weight change (left panel) or the sagittal abdominal diameter (middle panel), and between the body weight change and the sagittal abdominal diameter (right panel), were investigated. The linear correlation coefficient r (Spearman) is calculated for each panel. [file 12943_2024_2073_MOESM5_ESM.tiff]

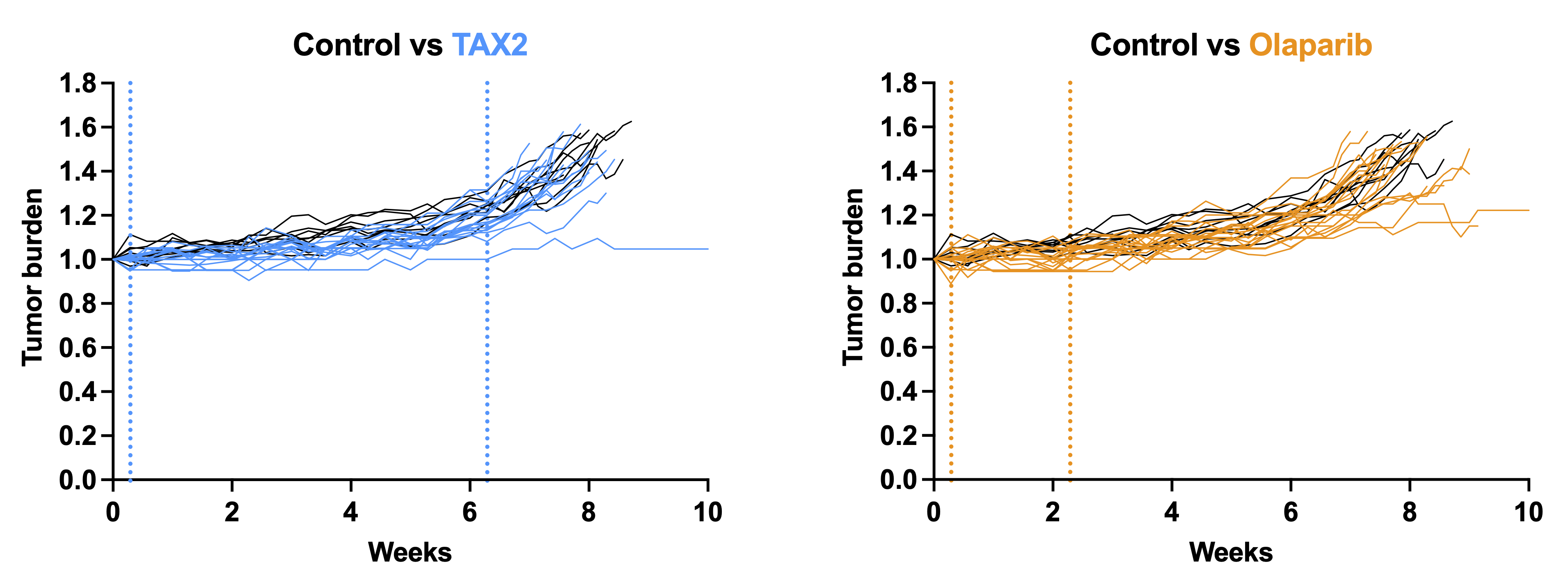

Supplement: Supplementary file 6 — Additional file 6: Suppl. Figure 6. C57BL/6 J female mice were IP inoculated using ID8 Trp53−/− Brca2−/− ovarian carcinoma cells, and then treated with vehicle (control, black), olaparib (orange), or TAX2 (blue) as detailed for Fig. 6. Individual tumor burden is represented. Dotted lines represent TAX2 (blue) or olaparib (orange) treatment duration. Mice without carcinosis at sacrifice were eliminated as described in the experimental procedure. [file 12943_2024_2073_MOESM6_ESM.tiff]

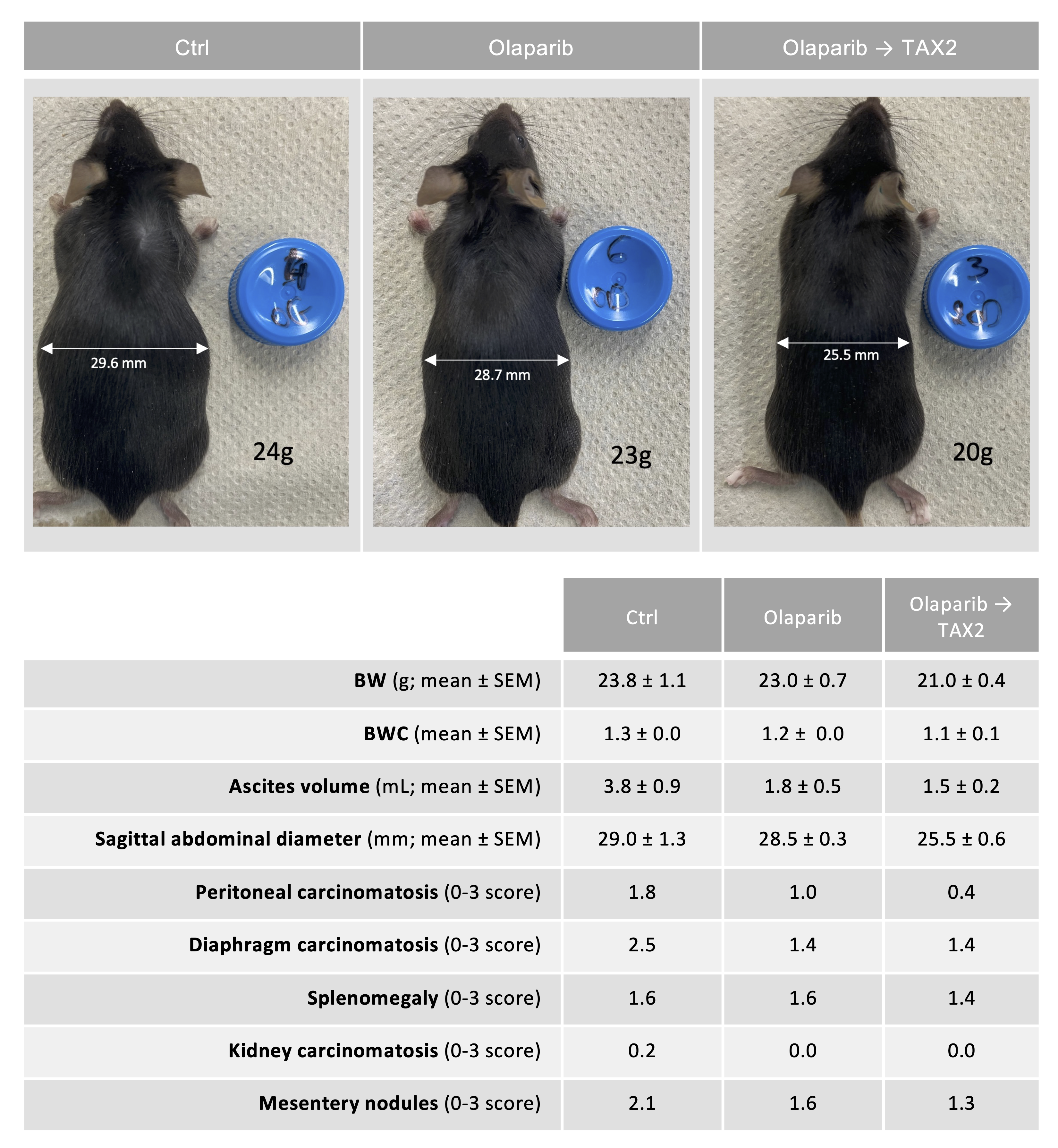

Supplement: Supplementary file 7 — Additional file 7: Suppl. Figure 7. Additional data illustrating the experiment described in Fig. 6c, 6 weeks after inoculation with tumor cells and administration of TAX2 at a 30 mg/kg BW dose, including photographs of mice showing abdominal distension [file 12943_2024_2073_MOESM7_ESM.tiff]
